# Gut Biofactory—Neurocompetent Metabolites within the Gastrointestinal Tract. A Scoping Review

**DOI:** 10.3390/nu12113369

**Published:** 2020-11-01

**Authors:** Karolina Skonieczna-Żydecka, Karolina Jakubczyk, Dominika Maciejewska-Markiewicz, Katarzyna Janda, Karolina Kaźmierczak-Siedlecka, Mariusz Kaczmarczyk, Igor Łoniewski, Wojciech Marlicz

**Affiliations:** 1 Department of Human Nutrition and Metabolomics, Pomeranian Medical University in Szczecin, 71-460 Szczecin, Poland; karzyd@pum.edu.pl (K.S.-Ż.); karjak@pum.edu.pl (K.J.); dmaciejewska.pum@gmail.com (D.M.-M.); katarzyna.janda@pum.edu.pl (K.J.); 2 Department of Surgical Oncology, Medical University of Gdansk, Smoluchowskiego 17, 80-214 Gdańsk, Poland; leokadia@gumed.edu.pl; 3 Department of Clinical and Molecular Biochemistry, Pomeranian Medical University in Szczecin, 70-111 Szczecin, Poland; mariush@pum.edu.pl; 4 Department of Gastroenterology, Pomeranian Medical University, 71-252 Szczecin, Poland; 5 The Centre for Digestive Diseases Endoklinika, 70-535 Szczecin, Poland

**Keywords:** microbiome, tryptophan, neurotransmitters, short chain fatty acids, brain

## Abstract

The gut microbiota have gained much scientific attention recently. Apart from unravelling the taxonomic data, we should understand how the altered microbiota structure corresponds to functions of this complex ecosystem. The metabolites of intestinal microorganisms, especially bacteria, exert pleiotropic effects on the human organism and contribute to the host systemic balance. These molecules play key roles in regulating immune and metabolic processes. A subset of them affect the gut brain axis signaling and balance the mental wellbeing. Neurotransmitters, short chain fatty acids, tryptophan catabolites, bile acids and phosphatidylcholine, choline, serotonin, and *L*-carnitine metabolites possess high neuroactive potential. A scoping literature search in PubMed/Embase was conducted up until 20 June 2020, using three major search terms “microbiota metabolites” AND “gut brain axis” AND “mental health”. This review aimed to enhance our knowledge regarding the gut microbiota functional capacity, and support current and future attempts to create new compounds for future clinical interventions.

## 1. Introduction

The gastrointestinal microbiota play a prominent role in maintaining human health. A very recent report demonstrated that a certain gut microbiome composition correlates with multiple disease markers [1]. Among the pleiotropic role of the digestive tract microbiota, the fermentative and thus metabolic functions deserve attention. First, gut microbes ferment food particles, which consequently makes them an anaerobic bioreactor of multiple tasks, involved in digestion of polysaccharides, synthesis of vitamins, short chain fatty acids (SCFAs), and polyamides. Some of these molecules possess neuroactive competences [2]. Of importance, some of microbiotas’ metabolic functions are common (e.g., acid and SCFAs production), while others are either species- (e.g., vitamin synthesis, bile salt metabolism, or enzymatic activity), or strain-dependent (e.g., immunological and endocrinological effects or production of specific bioactive molecules) [3].

Selected bacterial genera could synthesize neurotransmitters, such as gamma–amino butyric acid (GABA), serotonin, catecholamines, and histamine, which all together affect the central nervous system (CNS) via the enteric nervous system (ENS) and enterochromaffin cells [4]. Microbially produced vitamins and pivotal molecules act as cofactors of enzymes and maintain proper neural signaling. SCFAs, which predominantly act as trophic molecules, can affect the CNS. Although the discovery of specific receptors allowed for unraveling their neuroactive potential, more studies are needed to elucidate the exact role of SCFAs within the ENS and CNS. Other functions of the gut microbiota include bile acid biotransformation involved in N Methyl D Aspartate (NMDA) and GABA signaling routes. Gut bacteria can also degrade food derived tryptophan. Several tryptophan catabolites have been shown to bind to the aryl hydrocarbon receptor (AHR) located in the immune cells to mediate the innate and adaptive immune response, thus contributing to neuropsychiatric disorders of immune-related origin [5,6,7]. More recently, gastrointestinal (GI) microbiota were found to be involved in AHR expression within the GI nerves of the distant location, regulating, among others, gut peristalsis [8].

Of relevance, the alterations in the secretion of microbial derived metabolites have been described in patients who are mentally ill. For example, in patients with major depressive disorder (MDD), lower abundance of bacteria involved in SCFA production was described [9,10,11,12]. Moreover, microbial alterations that result in increased putrefaction have been implicated in the pathogenesis of autism spectrum disorders [13]. With the advent of novel techniques, such as metagenomics and metabolomics [14,15], capable of studying host-microbial metabolism, the link between gut bacterial metabolites and mental illness has been established. Therefore, in this scoping review, we aimed to update the readers on this important topic and present the currently available knowledge.

## 2. Methods

The methodology for scoping reviews by Arksey and O’Malley [16] with updates [17] was adopted for the present study. The major five key phases were as follows: (1) Identifying the research question: “what is known about the neuroactive activity of the gastrointestinal microbiota metabolites?”. We chose such a broad question with the purpose to identify and cover as many aspects of this topic as possible. (2) Identifying relevant studies: PubMed and Embase databases were searched using the following key words: microbiota metabolites, neuroactivity, nervous system, and psychiatry. We also conducted manual search of references in eligible reviews describing the impact of gut microbiome metabolites (overall) on the functions of central nervous system. We searched for studies in English, with no time restrictions regarding publication time. (3) Study selection: we selected studies conducted in both animals and humans, which provided mechanisms on the mode of action of particular neuroactive metabolites. The study selection was performed by the first and senior authors. This step was conducted over the period of two weeks. (4) Charting the data: we abstracted data on the study protocol (sample size, intervention—if applicable) and main outcomes referring to neuroactivity of microbially produced molecules. (5) Collating, summarizing, and reporting the results: we organized the data thematically according to 11 types of metabolites. We aligned the body of manuscript with the information on the most recent data on gut–brain axis (GBA) structure (including intestinal stem cells and enteric nervous system (ENS)) followed by a description of neurochemical mechanisms within the GBA and a summary of practical implications in diagnosis and therapy.

## 3. Gut Brain Axis—Complex Interplay between Gut Epithelium, Enteric Nervous System, and Central Nervous System

The gut and the brain are connected to one another through the gut–brain axis. The communication is of neural type—via the vagal nerve—but signaling within the GBA involves also the circulatory system with neurotransmitters, hormones, cytokines, and bacterial metabolites [18]. The intestinal homeostasis depends on complex interactions between multiple components of the gut/intestinal barrier (GB), including the following: (i) gastrointestinal microbiota; (ii) gut epithelial cells, (iii) gut endothelial cells; (iv) lymphatic vessels; and (v) ENS.

The small bowel villus is paved with a single layer epithelium responsible for host protection, absorption of vital nutrients, and exclusion of pathogens from the GI lumen. The crypt-villus unit is an important component of GB. The gut–brain communication is mediated via neural circuits and blood/bone marrow derived cells in their microenvironment. The source of mature epithelial cells is continuously dividing intestinal stem cells. The intestinal epithelium contains lgr5 (+) positive crypt base columnar cells (CBCs), with self-renewal potential and capability of producing more mature absorptive and secretory progenitors [19]. Another type of cell positioned in the intestinal crypt between the stem cell and progenitor zone has been named +4 cells [20].

Absorptive progenitors turn into mature enterocytes and M (microfold) cells. The intestinal epithelium is formed mainly by enterocytes with a major role of nutrients and water absorption. M cells overlie lymphoid follicles (Peyer’s patches) containing mononuclear cells as well as T and B cells. M cells protrude into and sense gastrointestinal lumen transporting signals to lymphoid cells [21].

The density of microfold (M) cells in ileum Peyer’s patch (PP) follicle-associated epithelia (FAE) is regulated by nociceptors, which also maintain levels of segmented filamentous bacteria (SFB), a gut microbe residing on ileum villi, and PP FAE that mediates resistance to *Salmonella* infection [22]. Secretory progenitors give rise to Goblet cells, Paneth cells, enteroendocrine cells, and Tuft cells. Goblet cells secrete protective mucus layer spanning the epithelium. The alterations of Goblet cells have been described in intestinal infections, cystic fibrosis, inflammatory bowel diseases, and some cancers [23,24,25,26]. Paneth cells are involved in signal transduction and columnar division, protecting intestinal stem cells at the bottom of the crypt. The proliferation, differentiation, and maturation of other gastrointestinal epithelial cells are also partly regulated by Paneth cells as well as fibroblasts, pericytes, myofibroblasts, smooth muscle, and neural cells. The pathology of Paneth cells has been described in neonatal intestinal necrosis (NEC), GI infections, and Crohn’s disease.

Enteroendocrine cells (ECs) comprise about 1% of the epithelium, release numerous hormones (e.g., cholecystokinin, serotonin, ghrelin, somatostatin) into the circulation, and create a structural/functional unit with vagal nerve. These cells play a role in gut–brain signaling and are involved in the regulation of the endocrine system [27]. Bellono et al. [28] have shown that ECs express specific chemosensory receptors, are electrically excitable, and modulate serotonin-sensitive primary afferent nerve fibers via synaptic connections, enabling them to detect and transduce environmental, metabolic, and homeostatic information from the gut directly to the nervous system [28]. EC alteration critically affects the physiological and homeostatic function of the gastrointestinal tract.

Tuft cells (also known as brush cells) are rare cells, which release opioids and immune mediators involved in anti-parasite intestinal defense and pancreatic cancer biology [29]. Mesenchymal cells secrete various ligands involved in division and maturation of cells, shaping the gut barrier elements (epithelium, endothelium, and smooth muscle cells). Mesenchymal cells are also involved in the maintenance of intestinal stem cell and integrity of intestinal epithelium. Various ligands secreted by epithelial cells also affect the mesenchyme and this bidirectional cross-talk forms the basis for complex interplays among different compartments of the intestinal barrier [21]. Blood circulating gut metabolites have been linked to the immune system with respect to microbial-associated molecular patterns. Ultimately, they are able to activate immune cells (e.g., macrophages, neutrophils, and dendritic cells) with the ability to synthesize inflammatory mediators crossing the blood brain barrier (BBB), where they have the potential to alter the physiological activity of neurons and microglia, acting as physical stressors [30,31].

These cellular components of GB interact with ENS. The myenteric plexus, localized between the longitudinal and circular muscle layers, regulates gut motor peristalsis and the submucosal plexus, above the circular muscle layer at the mucosa, coordinates intestinal secretory activity. Within intestinal plexuses, intrinsic (nerves from neurons and glia with cell bodies within the intestine) and extrinsic (neurons of the parasympathetic and sympathetic branches of the peripheral nervous system, dorsal root and nodose neurons, and vagal nerve) ganglia surrounded by glia communicate with other cellular components of the gut barrier [32].

It has already been found that the ENS regulates, among others, intestinal peristalsis and provides adequate digestion and fermentation of foods. The ENS regulates the feelings of hunger and satiety, and the perception of abdominal pain [33]. Conversely, it has been found that the gut microbiota have neuroactive and immunocompetent capacities, playing critical roles in shaping the key brain region structures. Furthermore, intestinal microbiota are essential for regulation of the neural activity and tissue plasticity, undisturbed transmission of nerve signals, and expression of neurotrophins [34].

## 4. Gut Microbiota and Its Neurochemical Activity

The importance of the gut microbiota metabolic potential has been recognized from an evolutionary point of view. Nevertheless, recent data have emerged to show the microbiota neurochemical activity [35]. It was elegantly reported that some of the bacterial genera have the ability to synthesize neurotransmitters, for instance, GABA, serotonin, catecholamines, and histamine, which all together reach the CNS via enterochromaffin cells and the ENS [4,28]. Microbially produced vitamins acting as enzyme cofactors and pivotal molecules, maintaining proper neural signaling, possess neurotrophic properties. With the aid of specific enzyme apparatus in bacterial cells, complex carbohydrates and glycans (e.g., mucin or oligosaccharides present in human milk) are broken down [36]. As a result of intestinal fermentation [37], SCFAs (acetate, propionate, and butyrate) are synthesized. Other compounds produced by intestinal bacteria are lactate, ethanol, succinate, valerate, capronate, isobutyrate, 2-methylbutyrate, and isovalerate. SCFAs predominantly act as trophic molecules. In this context, butyric acid is the most important, as it supplies enterocytes with energy [38]. With the discovery of SCFAs’ receptors within the CNS, their neuroactivity has also recently been acknowledged. Other functions of the gut microbiota comprise bile acid biotransformation involved in NMDA and GABA signaling routes. Gut bacteria have the ability to degrade tryptophan derived from foods. Several tryptophan catabolites were shown to bind to the AHR located in immune cells to mediate innate and adaptive immune responses, thus contributing to neuropsychiatric disorders with immune-related origin. Regarding immunity, the aforementioned SCFAs can drive myelopoiesis in the bone marrow, a process that initiates before birth. Butyrate produced by *Clostridia* activates the expression of transforming growth factor beta (TGF-β) in epithelial cells and epigenetically regulates gene expression by inhibiting histone deacetylases, which results in increased acetylation of histones in non-coding forkhead box P3 (FoxP3) sequences [39]. Interestingly, cells expressing Foxp3 play a major role in immune homeostasis.

Numerous studies have confirmed the existence of specific alterations in the intestinal microbiota composition during the course of mental disorders [4,35,40,41,42], including depression, schizophrenia, autism spectrum disorders, and Parkinson’s disease, among others. Importantly, the coexistence of disorders of gut–brain interaction (DGBIs) and CNS diseases has been well documented. It has been estimated that at least 36.5% of patients with DGBIs express the symptoms of mental disorders, with the most common overlap of functional constipation (60%) compared with those with functional dyspepsia (52.4%) or bloating (47.6%). Additionally, panic anxiety disorder is often diagnosed in individuals suffering from gastrointestinal diseases [43,44]. Meanwhile, psychological treatments have been shown to counteract the symptoms of irritable bowel syndrome (IBS) [45]. Such treatments are mainly maintained through neuromodulators or psychotropic drug administration [46], which have antimicrobial properties [47]. Further, such treatment might stand for a poor metabolic outcome in selected patients [48,49]. Currently, it remains unanswered whether the microbiota alterations observed in mental illnesses with concomitant metabolic malfunctions are involved in the genesis of these disorders or solely their consequence.

Apart from compositional disturbances within the gut microbiota, skewed metabolic functions have also been suggested. These are the predictions made upon the basis of sophisticated software, e.g., Phylogenetic Investigation of Communities by Reconstruction of Unobserved States (PICRUSt) and exact metagenomic analyses. For instance, a PICRUSt2 analysis in patients with depression demonstrated that MDD phenotype might be linked to pathways involved in vitamins (folate and thiamine), lipopolysaccharide (LPS), and long-chain fatty acids biosynthesis. The pathways observed in the study of healthy subjects were related to fermentation of SCFAs, phospholipid biosynthesis, nucleic acid metabolism, and aliphatic amino acid [50]. In another study, the authors developed an analytical framework for targeted profiling and interpretation of metagenomic data describing metabolic pathways, which may include neuroactive compounds, and selected 56 gut–brain modules (GBMs), corresponding to a single neuroactive compound production or degradation process, and showing a tendency of decreased glutamate degradation potential in patients suffering from depression. [51]. A study by Lai et al. [52] demonstrated that tryptophan (Trp) metabolism and biosynthesis were altered in patients with depression. Another clinical trial [53] revealed that, after probiotic ingestion, Trp catabolites’ (TRYCAT) concentrations were altered. The ratio of 3 hydroxykynurenine (3HKYN)/kynurenine increased, while the kynurenic acid (KYNA) concentration decreased. Additionally, indole with derivatives also originating from Trp have also been demonstrated to be ligands for AHR and, via immune-related pathways [54] and restorative neurogenesis timing [55], linked to the pathogenesis of depression [56,57]. A very recent metagenome-wide study of the microbiome in patients with schizophrenia reported that differences in SCFA synthesis, tryptophan metabolism, and synthesis/degradation of neurotransmitters are of major importance in this entity [58]. In such patients treated with olanzapine, clustering the study cohort according to *Prevotella* genus consequently predicted differentially abundant pathways, including carbohydrate metabolism, by means of linear discriminant analysis with effect size [59]. The same year, fecal microbiota transplants (FMTs) from patients with schizophrenia led to schizophrenia-associated behaviours in donor mice and were shown to alter the concentrations of glutamate, glutamine, and GABA in the hippocampus of recipient rodents [60]. In patients with autism, altered microbiota composition was accompanied by reduced tryptophan levels in plasma and urine [61] and high serotonin concentrations [62].

**Figure 1:**
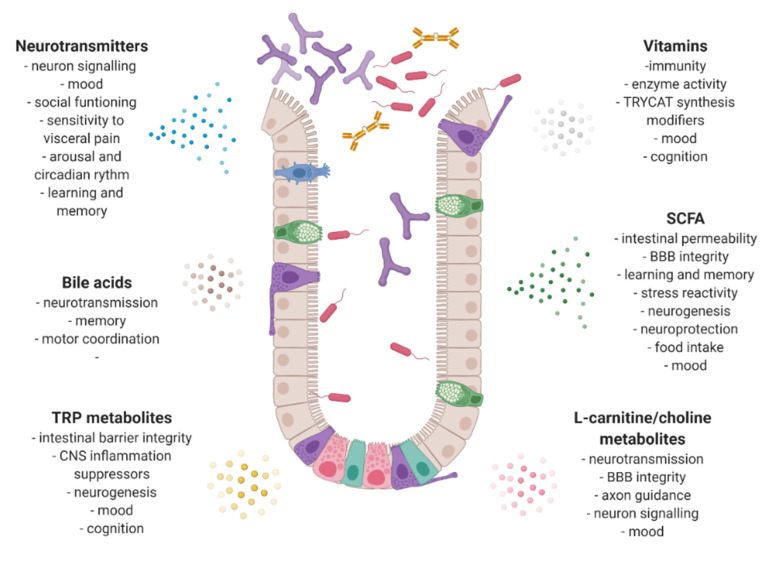
Gut microbiota metabolites and their neuroactive potential. SCFA—short chain fatty acid; TRYCAT—tryptophan catabolites; TRP— tryptophan; CNS—central nervous system; BBB—blood brain barrier. Created with Biorender.com.

Overall, gut microbiota alterations in neuropsychiatric entities result in skewed metabolites production (Figure 1), which all together may evoke systemic low-grade inflammation and an imbalanced neural signaling via the brain–gut–microbiota axis. Both gut compositional analyses and its functional dysregulation may contribute to mental disorder pathogenesis, supporting the hypothesis of a pathological process of bidirectional communication between the gut and the brain. In this way, microbially produced molecules are surrogate markers of susceptibility to express certain clinical phenotypes and the response to pharmacotherapy [63].

## 5. Bacterial Neurotransmitters

It has been demonstrated, predominantly in in vitro studies, that neurotransmitters including GABA, serotonin, catecholamine, and histamine are produced within the gut (Table 1).

GABA is an inhibitory neurotransmitter in humans, linked with skewed functions of the CNS and ENS when synthesised abnormally [66,67]. Germ-free animals diminish the production of GABA, as its concentration was lowered in stool and blood [68]. GABA derived from gut were shown to cross the BBB [69,70] and its synthesis depends on antibiotic exposure [71] and on diet, as high fat food intake lowered the GABA levels in the rat prefrontal cortex. Peripherally, GABA acts to diminish visceral pain [72]. Additionally, that concentration was linked to lowered abundance of the GABA producer (i.e., *Bacteroides*), thus associated with depressive-like behaviour [73].

Increased GABA levels in blood collected from patients with MDD have been documented [74] and gut-derived GABA has been confirmed to act within the GBA [75]. Recently, Strandwitz et al. [65] demonstrated that the left anterior medial frontal cortex functionally connected to the left dorsolateral prefrontal cortex (inactive within the course of depression) was inversely correlated with *Bacteroides count.* Consequently, the intake of GABA producing probiotic strains resulted in improved anxiety- and depression-related mood [76,77] along with reducing sensitivity to visceral pain, but only in cases of GABA overexpression [78]. Nevertheless, we should note that GABA signalling is indirectly mediated by gut microbiota via the vagus nerve only [79].

A cohort study named Flemish Gut Flora Project (FGFP) [51] focused on skewed glutamate pathways, in particular glutamate degradation to crotonyl-coenzyme A and acetate and the GABA shunt pathway, although the association disappeared after testing for multiple hypotheses. Alterations in GABA signalling have been associated with anxiety and depression. Moreover, it has been stated that faecal microbiota transplantation (FMT) from patients with schizophrenia to germ-free (GF) rodents influenced the glutamate glutamine-GABA cycle, resulting in increased startle responses and locomotor hyperactivity [60]. This study also reported elevated glutamine levels in the serum and hippocampus and decreased glutamate levels in the stool and hippocampus in transplanted animals compared with the controls. Further, glutamate synthase was more active in the digestive tract of patients with schizophrenia than in healthy controls and its high activity was associated with altered gut microbiota taxonomies associated with gut IgA levels [80].

Dopamine (DA) is a pivotal reward-motivated behaviour mediating neurotransmitter and notably a precursor of noradrenalin and adrenaline involved in waking state, cognition, and behaviour [81]. DA and adrenaline were synthesised in low concentrations in GF rodents and expressed in biologically inactive and conjugated forms [82]. In contrast, in cases of elevated DA production, the rate of pathogenic bacteria growth (e.g., *Escherichia coli* O157:H7, *Klebsiella pneumoniae, Pseudomonas aeruginosa, Enterobacter cloacae, Shigella sonnei*, and *Staphylococcus aureus*) increased [83,84] along with elevating its virulence [85]. Although such data indicate that catecholamines cannot cross the BBB, tyrosine (Tyr), which is a precursor of DA, was elevated in the cerebrum of re-colonised GF mice compared with GF counterparts, implying that the gut microbiota affect the enzymes transforming Tyr into DA. Thus, they indirectly affect the CNS function [68,86]. Of note, depletion of gut microbiota in mice following antibiotic exposure enhanced the turnover rate of cocaine linked to increased activity of DA receptor, which was further reversed by SCFAs supplementation [87].

The synthesis 3,4-dihydroxyphenylacetic acid (DOPAC), which is a DA metabolite, was positively correlated with the social functioning score in participants of the FGFP [51]. Interestingly, in this population data set, DOPAC synthesis potential was linked to enterotype distribution (i.e., it was lower in the *Bacteroides* enterotypes 1 and 2) and *Prevotella* community types compared with *Ruminococcaceae*-enterotyped samples. Of relevance, the DOPAC synthesis potential was most strongly associated with the relative abundance of *Coprococcus*, a bacterial genus that is reported to be correlated with the mental and physical ‘Research and development’ scores [51]. At last, the low levels of this metabolite were reported to be a biomarker in Parkinson’s disease [88].

Histamine was also shown to be produced in the intestine and affects arousal, cognition, reward and memory, and circadian rhythm [89,90]. Importantly, when the histamine receptors were blocked, intestinal barrier integrity was damaged as a result of diminished mucus secretion [91]. In contrast, histamine production by some probiotic strains was shown to lower the production of proinflammatory mediators like tumour necrosis factor-a in myeloid progenitor cells [92,93], a mechanism also involved in *Yersinia enterolica* infection [94]. The production of histamine was additionally reported as critical for colonic homeostasis, as the amine was demonstrated to regulate the NLRP6 inflammasome. Histamine significantly suppressed the secretion of interleukin (IL)-18 as a result of reduction in NLRP6 inflammasome assembly [95].

Finally, serotonin (5-hydroxytryptamine [5-HT]), which is predominantly produced by enterochromaffin cells [96], plays a pivotal role during neuronal differentiation and migration, axonal outgrowth, myelination, and synapse formation [97]. In GF mice, its production is low [98], and although its production by gut microbiota has not been confirmed, a systemic serotonin pool is mediated by Trp metabolism [99].

## 6. Short Chain Fatty Acids

SCFAs are saturated fatty acids that have one to six carbon atoms in the hydrocarbon chain [100]. These are fermentation products that form in the intestines when the undigested fermentable saccharides, including resistant starch, non-starch polysaccharides, and lignins, commonly known as dietary fibres, are broken down by anaerobic intestinal bacteria. Fibre is the major source of SCFAs for mammals, including humans [101,102]. However, SCFAs can also arise from the fermentation of amino acids or proteins. The products are branched chain fatty acids, H_2_, CO_2_, CH_4_, phenols, and amines. Some of these (e.g., phenolic compounds, biogenic amines, and ammonia) may cause damage to the intestinal epithelium and induce inflammation in situ. Therefore, a diet rich in fermented carbohydrates save proteins is proposed as a common dietary intervention [101].

SCFAS are produced at daily amounts of 500–600 mM [100] with acetic (C2), propionic (C3), and butyric (C4) acids predominantly synthesized. The molar ratio of these acids is 60:20:20; however, the part of the intestine, diet, age, and health status may modify that significantly [100]. Butyrate is subjected to the greatest modulation in terms of concentration during life and acetate represents more than half of the SCFAs pool in the faeces [101]. Acetic acid might also be produced by *Acetobacter* [103,104,105] and to some extent in the liver, as a result of ethanol oxidation and during the ketogenesis and oxidation of free fatty acids [102].

Butyric acid is mainly produced by the following bacterial genera: *Clostridium, Eubacterium,* and *Fusobacterium* [106], with the most effective producers including *Clostridium leptum, Roseburia* spp*., Faecalibacterium prausnitzii,* and *Coprococcus* spp [107]. Propionic acid is synthesised by *Bacteroidetes* and *Propionibacterium* [108]. Acetic acid is considered to be produced by *Bacteroidetes* and further metabolised by *Firmicutes* to create butyrate and propionate [109]. Parallelly, butyric or propionic acid might be re-metabolised to acetic acid with the association of *Acetobacterium, Acetogenium, Eubacterium,* and *Clostridium* [101].

SCFAs bind to corresponding receptors and mediate interactions within the microbiota–gut–brain axis [100]. To date, the best known SCFAs receptors are G protein coupled receptor 43 (GPR43) and GPR41, recently entitled as free fatty acid receptor 2 (FFAR2) and FFAR3, respectively. Receptors are activated by SCFAs anions, such as acetate, butyrate, propionate, and formate [110]. FFAR2 interacts mainly within the enteroendocrine L, immune, and vascular cells [111,112], while FFAR3 has been identified in the colon, kidneys, sympathetic nervous system, and blood vessels [113,114]. The evidence that fatty acid transport proteins are expressed within the human brain microvessel endothelial cells has proven that these molecules, including butyrate [115], have the potential to enter the environment of the brain [116,117]. Such observations were also made historically in an experiment with carbon-14 labelled SCFAs injected into the carotid artery to be uptaken by the brain [118]; however, small amounts were injected according to each human body [119]. Likewise, there is evidence that FFAR3 is expressed in the rat brain tissue and sympathetic ganglia [120]. The FFAR3 receptor has been found to bind propionate, which further acts on the periportal afferent neural system and peripheral central nervous areas to induce gut gluconeogenesis [121]. Furthermore, an in vitro study demonstrated that a human cerebrovascular endothelial cell line with FFAR3 expression exposed to LPS lost its integrity. When their barrier was treated with 70 kDA fluorescein isothiocyanate conjugated dextran, its permeability increased along with decreased trans-endothelial electrical resistance. Surprisingly, when the cell line was treated with different SCFAs, the altered parameters of BBB integrity improved, with propionate being the major molecule involved. The metabolite additionally restricted non-specific microbial infections via a CD14-dependent mechanism and protected the BBB from oxidative stress via nuclear factor erythroid 2–related factor 2, showing its potential to control oxidative damage within the CNS [122].

Consequently, when describing the systemic effect of SCFAs, we should consider the association with mental disorders. A number of studies on the effects of SCFAs on the nervous system and the behaviour proved that low SCFA levels were positively correlated with mental disorders, including depression, Alzheimer’s (AD) or Parkinson’s disease, and others [100]. Jiang et al. [123] reported that *Lachnospiraceae* and *Ruminococcaceae* families, within the phylum Firmicutes, were diminished in patients with MDD compared with the controls. A study [124] reported the expression of various *Lachnospiraceae* and *Ruminococcacea* species, known to participate in the breakdown of carbohydrates into SCFAs. Consequently, a decrease in their counts resulted in SCFA production decrease, which caused intestinal barrier dysfunction [38,125]. More recently, a pilot study [126] focusing on drug-naïve Chinese patients with schizophrenia observed decreased SCFA-producing bacteria, namely *Faecalibacterium* and *Lachnospiraceae* genera. Other studies have also shown that the intestinal microbiota of patients suffering from depression produce smaller amounts of SCFAs content compared with healthy participants [127,128]. *Coprococcus catus* could produce propionate and butyrate, two SCFAs [104], and its abundance is associated with better quality of life [51]. Importantly, the intake of *Clostridium butyricum* MIYARI 588, producing SCFAs efficiently and integrally during an antidepressant therapy, improved the emotional state of patients with depression [129]. However, clarification of the role of SCFAs in depression requires further studies. Kelly et al. [130] did not observe differences in SCFAs’ concentration in faeces between depressed and healthy subjects. Moreover faecal microbiota transplantation from patients with depression to rats after sterilization of gastrointestinal tract led to changes in behavior and metabolic parameters, significant for depression. Surprisingly, this intervention also caused an increase in faecal SCFAs’ concentration. This means that FMT alone can increase the SCFAs’ concentration in stool, independently of clinical outcome. Conversely, in a mouse AD model, administration of butyrate resulted in improved memory and effective learning [131], an effect that might be related to SCFAs interference with amyloid-β peptides, counteracting neurotoxic oligomers assembly [132]. In Parkinson’s disease [133], butyrate supplementation improved motor function and reduced DA deficiency in patients potentially via inhibiting alpha synuclein-induced DNA damage [134,135], which is also caused by oxidative stress [136]. Animal models of mania were utilised to confirm that sodium butyrate might improve amphetamine induced hyperactivity and respiratory chain complexes activity to finally restore non-maniac behaviour [137,138]. Overall, butyrate presents high potential to affect brain physiology [139]. The study in patients with autism spectrum disorders (ASDs) demonstrated elevated abundance of *Proteobacteria* and *Desulfovibrio*, both producing potentially toxic metabolites [140,141]. Moreover, propionate has been used as the major external factor, which is able to express ASD-like phenotype in animals despite the administration route. As a result, microglia activation and neurotoxic mediators production occurred, resulting in skewed neuro-behaviours, including repetitive and impaired social interaction [142] The aforementioned effects are, at least partly, attributed to SCFAs’ ability to regulate the body’s immune response. Butyric acid plays a crucial role here, as it mediates histone deacetylase inhibition through GPR41, GPR43, and GPR109 receptors or through the binding of two butyrate molecules in the hydrophobic part of the enzyme, which is an example of the intracellular SCFA action [143]. Further, the secretory activity of macrophages associated with the production of pro-inflammatory cytokines IL-6 and IL-12 has been demonstrated to diminish the secretion of butyrate [144]. Additionally, the administration of butyric acid decreased the production of CXCL-2 and -3 by neutrophils, which resulted in reduced recruitment of leukocytes following immune stimuli [145].

Regulation of the immune response by SCFAs also manifests protection against autoimmunity. These bacterial metabolites activate T-regulatory (Treg) lymphocytes, which inhibit the elevated immune or auto-aggressive response directed against the body’s own cells. Studies have suggested that SCFAs initiate the transformation of naive TCD4 + (cluster of differentiation T cells) into regulatory cells [146,147], while others have reported that SCFAs stimulate Treg cells already presented in the colon [148]. A study [149] demonstrated that colonisation of the intestinal milieu in gnotobiotic mice with appropriate *Clostridium* strains, which are main butyrate producers, affected the multiplication and differentiation of Treg cells and stimulated the secretion of anti-inflammatory IL-10 and inducible T-cell co-stimulator. Moreover, microbiota and their metabolites were recently demonstrated to shape the immune phenotype of microglia. Antibiotic treatments, dysbiosis, and additional knock-out for *FFAR*2 receptors resulted in immature immunophenotype of these cells within the CNS, but were restored with SCFAs’ supplementation or recolonisation with the complex gut flora [150].

Finally, SCFAs have also been assigned an important role in regulating the body’s metabolic functions indirectly via the nervous system. These gut metabolites were demonstrated to increase the concentration of leptin and insulin, which are hormones that stabilize the feeling of satiety and are secreted by adipocyte and pancreatic cells [151]. SCFAs also affect the secretion of peptide YY (PYY) and glucagon-like peptide-1 (GLP-1) via the GPR43 receptor located in the endocrine L cells of the ileum and colon. PYY and GLP-1 are hormones responsible for the feeling of satiety, and stimulation of their production by SCFAs may result in reduced food intake [152]. Concerning the food intake amount regulation, acetic acid itself also plays an important role, which suppresses appetite by activating acetyl coenzyme A carboxylase and its effect on the regulatory neuropeptide expression in the hypothalamus [153].

## 7. Tryptophan Metabolites

Trp is an essential exogenous amino acid circulating with albumin or in free form. Trp transport through the BBB occurs only in free form via non-specific L-type amino acid transporters [154,155]. Trp is an essential amino acid for humans, which contributes to controlling emotional states (i.e., happiness, well-being, cellular aging processes, and energy production) through its conversion to serotonin or nicotinamide adenine dinucleotide (NAD+) [154].

Moreover, it is a precursor of metabolic pathways, such as the kynurenine (KP) (contributing to the production of various neuroactive compounds) and serotonin pathways [154,156]. Approximately 90% of the Trp catabolism by KP results in the production of metabolites including KYNA and quinolinic acids [154], acting on the glutamate receptor [157]. Other intermediates are KP, 3HKYN, 3-hydroxyanthranilic acid (3HAA), and picolinic acid [158]. The rate of Trp metabolism along the KP depends on the expression of indoleamine 2,3-dioxygenase (IDO1) enzyme found in all tissues and tryptophan-2,3-dioxygenase acting in the liver [159]. IDO1 activity has been found to be elevated in the presence of cytokines and other inflammatory molecules (i.e., interferon γ and amyloids). KP metabolites play crucial roles in numerous neurodegenerative disorders. As shown in Table 2, different KP intermediate to amyotrophic lateral sclerosis and Alzheimer’s, Huntington’s, and Parkinson’s diseases compared with the controls [154]. Further, increased KP activity can lead to disorder through the production and accumulation of neurotoxic intermediates, such as quinolinic acid [154,160,161,162], affecting anxiety, depression, cognitive performance, and behaviour [163].

Bacteria are able to directly metabolise Trp, and thus change its availability and produce catabolites affecting the signaling within the GBA [154,165,166,167]. When mice were infected with *Trichuris muris*, the rodents were anxious and the plasma kynurenine/Trp ratio increased. When the mice were vagotomised before the infection, the results were similar, implicating that the vagal route was not involved in spreading the effects of infection. Anti-inflammatory agents and probiotic *Bifidobacterium longum* restored the behaviour, although in different manners [168]. IDO1 can additionally make Trp inaccessible to the intestinal bacteria by transforming it into potentially antimicrobial intermediates, such as KP, which further might deplete the gut ecosystem [154]. Gut bacteria may also produce some compounds, such as hydrogen peroxide, which inhibit IDO1 [169,170,171]. The ingestion of *Lactobacillus johnsonii* by rats reduced the level of IDO1 and kynurenine in the serum. Similarly, the cell-free suspension of the probiotic reduced IDO1 activity by 47% in HT-intestinal epithelial cells [161,169,171]. In contrast, transmission of *Bifidobacterium infantis* into rodents increased the Trp levels and the ratio of KYNA/kynurenine, which was also associated with reduced IDO1 activity [172].

In vivo studies have shown that, in GF animals, kynurenine activity and the kynurenine/Trp ratio were reduced, but were restored after microbiota administration [173,174,175]. KP disorders present important neurodevelopmental implications. Prenatal inhibition of the KP in rodents has been shown to cause changes in the hippocampal neuronal morphology, differences within the cerebral cortex, and changes in cerebellar protein expression that persist into adulthood [163,176,177]. Moreover, increased synthesis of KYNA in animals caused neurochemical and cognitive deficits in adulthood [178,179,180].

Intestinal microorganisms can metabolize the Trp, as a precursor to the synthesis of indole, melatonin, and serotonin, thus limiting this amino acid availability to the host [97]. Increased Trp metabolism has been associated with *Burkholderia, Pseudomonas, Ralstonia, Klebsiella,* and *Citrobacter* belonging to *Proteobacteria*. Consequently, a high abundance of *Proteobacteria* has been shown to be correlated with many diseases, including brain diseases [168,181]. *Burkholderia* and *Pseudomonas* affect the synthesis of kynurenine and quinolinate, which have been correlated with ASD phenotype. In contrast, *Akkermansia, Alistipes, Porphyromonas, Lactobacillus,* and *Staphylococcus* have been linked to the Parkinson’s disease aetiology owing to the production of indole and its derivatives [166]. Scientific research has shown the ability of some bacteria, *Enterococcus* and *Pseudomonas*, among others, for the production of serotonin in media rich in Trp [182,183]. Reducing the circulating Trp levels by the intestinal microflora affects serotonergic neurotransmission, thus affecting the central and intestinal nervous system functioning [97,184].

The intestinal microbiota affects the availability of circulating Trp and kynurenine, while KYNA and quinolinic acid do not cross the BBB in significant amounts. However, with intestinal microflora disorders, this can be modulated, as observed in GF mice [163]. Additionally, reducing Trp availability and concentration and increasing kynurenine and quinolinic acid concentrations limit the production of important neurotransmitters, such as serotonin in the brain [170,185]. The low serotonin levels are an important factor associated with depression, fatigue, and cognitive impairment [186,187]. Further, psychotropic drugs were shown to have antimicrobial effects [188], which could be linked to the ability of antidepressants (e.g., tricyclic drugs) to bind to the bacterial leucine transporter (LeuT) (i.e., the bacterial homologue of the neurotransmitter transporters) [189,190]. Trp indole metabolites, including indole-3-acetic acid and indole-3-pyruvate, may affect the CNS inflammation, microglia activation, or astrocyte function [54,57,163,191,192].

Metagenomic analyses based on shotgun sequencing share light on Trp metabolic pathways and their role in mental disorders. Lai et al. [52] analyzed the microbial Trp biosynthesis and metabolism pathway and found two and one lower KEGG orthologues (KOs) abundances in the Trp biosynthesis and metabolism pathways, respectively. Moreover KO abundance was correlated with MDD symptoms. Under physiological conditions, 5-HT cannot pass the BBB. Instead, Trp and 5- HT could pass the BBB, as precursors for the production of 5-HT [165]. Moreover, lower plasma Trp [193,194,195,196] concentrations and availability of 5-HT and its transporter (serotonin transporter) in the brain have been listed as key features in MDD pathogenesis [197,198,199,200,201]. Further, other studies have shown that Trp metabolism is linked to anxiety symptoms [202,203], while serotonin levels were positively correlated with anxiety and depression in patients with anorexia nervosa [204]. Importantly, indole and its derivatives, ligands for the AHR [205,206], are important for the intestinal immune balance [207,208], intestinal barrier permeability [209,210], and suppression of the peripheral CNS inflammation [54,211], all involved in MDD pathogenesis [51,63,212,213,214,215]. The data also showed that AHR deficiency may lead to the hippocampal neurogenesis failure [55,216], a process linked to MDD [217,218]. Thus, Di Giaimo et al. concluded that diminished AHR signaling may significantly modify the hippocampal neurogenesis [55]. Moreover, several bacteria are involved in Trp metabolism (e.g., *Enterococcus, Lactobacillus, E. durans*), facilitating the Trp biosynthesis pathway [219,220,221]. The data on the regulation of Trp metabolism (i.e., *Lactobacillus*) could convert the carbon source from sugar to Trp and produce AHR ligands, in order to further support the intestinal immune balance [207,222].

Probiotics can influence Trp metabolism. Recent probiotic studies [53,223] have demonstrated that the blood kynurenine concentration decreases and influences Trp metabolism after probiotic administration (*Lactobacillus plantarum* 299v and combination of *Lactobacillus helveticus* Rosell-52 and *Bifidobacterium longum* Rosell-175). Kynurenines have neurotoxic and neurodegenerative effects on the CNS and could play a significant role in MDD pathogenesis [58,224]. Zhu et al. [58] analyzed faecal metagenomes and identified 27 schizophrenia associated gut brain molecules among the several Trp metabolites. The results of previous animal study demonstrated that FMT from patients with schizophrenia who were medication-naive into specific pathogen-free mice could have influenced schizophrenia-like behavioural abnormalities and dysregulated kynurenine metabolism.

## 8. Vitamins

Experiments performed for many years in rodents (GF and conventional) and clinical studies have shown that gut microbiota has the ability to synthesize some vitamins, including vitamin K and B (e.g., biotin, cobalamin, folates, nicotinic acid, pantothenic acid, pyridoxine, riboflavin, and thiamine (vitamins B7, B12, B9, PP, B5, B6, B2, and B1, respectively)) [225,226].

Studies have indicated microbial species that can synthesize vitamins de novo (known as vitamin prototrophs) are largely used by others not producing these compounds (known as vitamin auxotrophs) [227,228]. B vitamins are, among others, important cofactors and coenzymes in several metabolic pathways. Moreover, recent studies have shown that B vitamins play an important role in maintaining immune homeostasis, also within the CNS [229,230]. Magnúsdóttir et al. [228] presented a comprehensive assessment of the possibility of vitamin B synthesis by human intestinal microbiome. The authors studied the genomes of 256 common intestinal bacteria in the context of their biosynthesis pathways for biotin, cobalamin, folic acid, niacin, pantothenate, pyridoxine, riboflavin, and thiamine. They concluded that only some genomes contained all eight pathways needed for biosynthesis of these vitamins [228,231]. The most commonly synthesized vitamins were riboflavin and niacin (166 and 162 potential producers, respectively). Concerning riboflavin and biotin, all bacteria belonging to *Bacteroidetes*, *Fusobacteria,* and *Proteobacteria* had the pathways necessary for their biosynthesis. *Firmicutes* and *Actinobacteria* strains proved to be much less effective in the synthesis of B vitamins [228].

Importantly, vitamins B2 and B6 are cofactors in kinurenine metabolism [232], like an active form of vitamin B6 (pyridoxal 5′-phosphate) being a cofactor for kynurenine aminotransferase and kynureninase or flavine adenine dinucleotide (an active form of vitamin B2) being a cofactor for kynurenine 3-monooxygenase (KMO) [232]. Of note, these vitamins need one another for their conversion into their bioavailable forms. It has been documented that supplementation with vitamin B6 led to increased KP enzyme activity with a decrease of the kynurenine levels [233]. Interestingly, *Lactobacillus plantarum* strains are known to synthesize vitamin B2 [234] and administration of *Lactobacillus Plantarum* 299v (LP299V) to healthy volunteers resulted in significantly increased faecal lactobacilli and bifidobacteria levels [235], which are well-known producers of vitamin B6 [236]. A study [53] observed an increased 3HKYN/kynurenine ratio in probiotics, reflecting KMO activity, but with no effect on plasma concentrations of 3HKYN and 3HAA. The activation of KMO towards NAD+ and adenosine triphosphate synthesis without elevated synthesis and storage of detrimental kynurenines might be the result of these crucial vitamin syntheses by LP299V. Finally, exposure to psychological stress and inflammatory state may elevate the utilization of kynurenine enzyme cofactors, resulting in increased production of detrimental TRYCATs [237,238].

## 9. Bile Acids

Cholesterol in hepatocytes is metabolized to primary bile acids (BAs) due to cytochrome P450 (CYP 450) enzymes [239], of which the most important are cholic (CA) and chenodeoxycholic acids (CDCA). Then, the primary BAs proceed inside the intestine, where they undergo further modifications owing to the activity of the intestinal microbiota. The products of this conversion are secondary bile acids. CA and CDAC are converted to lithocholic and deoxycholic (DCA) acids. Approximately 95% of BAs are effectively absorbed in the terminal ileum and transported to the liver via portal circulation [240].

BAs are an important part of cell signaling and are ligands for key nuclear receptor receptors, such as farnesoid X receptor (FXR) [241]. The latter is mainly expressed in hepatocytes and ileum, although it has also been found in the CNS [242]. It has been relatively recently discovered that FXR is also expressed in the brain, where it is primarily located in neurons of the cerebral cortex [243]. BAs can activate various nuclear receptors, including pregnane X, vitamin D, constitutive androstane, and glucocorticoid receptors, the majority of which are also expressed in the CNS [244]. BAs are also associated with receptors regulating neurotransmission. CA, DCA, and CDCA could inhibit the NMDA and GABA receptors. Interestingly, during in vitro studies, histaminergic neurons exposed to ursodeoxycholic acid blocked GABA signaling and postsynaptic transmission [245]. In vivo studies have shown that the FXR receptor removal interfered with memory and motor coordination and leads to changes in the regulation of GABA, glutamate, noradrenaline, and neurotransmission of serotonin [246]. BAs can also activate spinal neurons by a transient receptor potential ankyrin1 TRPA1-dependent mechanism involved in jaundice associated pruritus [247].

## 10. L-Carnitine/Choline Metabolites

Bacterial metabolites derived from nutrients contribute to the CNS function regulation, among them, choline substances, such as phosphatidylcholine, trimethylamine oxide (TMAO), and L-carnitine biotransformation, regulate neurotransmitter pathways and ensure continuity of the CNS metabolic pathways [248].

A diet rich in animal products, especially red meat, provides large amounts of L-carnitine and choline-containing compounds. These compounds are converted by the intestinal microbiome to trimethylamine. The latter is then converted to TMAO by liver enzymes. TMAO concentration has proved to be useful in diagnosing and forecasting the risk of cardiovascular disease [249]. Recent studies have shown that TMAO is detectable in human cerebrospinal fluids. It also appears that TMAO may disrupt the BBB by reducing the expression of tight junction proteins, such as claudin-5 and tight junction protein-1 [250]. Xu and Wang have created algorithms to help identify TMAO as a metabolite associated with various aspects of AD. They also found common genetic pathways underlying AD and TMAO biomarkers. The strongest association between TMAO and AD was observed for pathways related to AD, axon guidance, immune systems, neuron signaling, as well as lipid and protein metabolism [251]. Another study has revealed that TMAO could stabilize and modify aggregation of beta amyloid (Aβ), favouring and accelerating the transformation of the random Aβ peptide chain into the conformation necessary to create the fibrillar structure [252].

Regarding mood, in patients with MDD, predominantly in fluoxetine responders, reduced choline content within the hippocampus and basal ganglia was reported [253]. Similar results were provided for patients prior to electroconvulsive therapy [254], which is contradictory to results obtained in other trials recruiting patients with depression [255,256]. The mechanism, although still unclear, might involve the ability of choline metabolism alterations to skew DNA methylation in the brain, as evidenced in prenatal mice and offspring with neurodevelopmental deficiencies [257]. This is in line with another study reporting that, in a model of early-life stress, ingestion of choline and betaine, which served as methyl donors, was linked to reversal of the depression phenotype in offspring [258].

## 11. Diagnostic and Therapeutic Potential

Past reports have demonstrated that psychiatric entities are complex, with the pathophysiology not fully understood. The promising approach to discover the background of these diseases was genomics with the emphasis on genome wide associations studies that aimed to point to heritability as being the most crucial [259]. However, multiple loci with unknown function, and thus biological effect or genetic overlap between particular entities [260], state that the development for other ‘omics’ techniques has to be implemented in the neuropsychiatric research. Other modern state-of-the-art techniques include transcriptomic, proteomics, and metabolomics, with the latter being considered to have closest to phenotype [261]. Metabolomics can detect small biological molecules and metabolites, which, unlike genes and proteins, are of a wide nature. Their production is strongly dependent on genetic and environmental factors. Further, their synthesis is cell-specific. Therefore, all together, the concentration of metabolites provides information on nutrition aspects, exposition to medications, other xenobiotics, stress [262], and internal pathological states [263] within the human body. 

Metabolomics have only recently started to be implemented in neuropsychiatric science [264,265]; however, as a vast majority targeted the CNS tissues and fluids, they were conducted in animal models [266]. For instance, amino acid, energy, and lipid metabolisms and disturbances in neurotransmitters were demonstrated to be skewed in a rat model of depression, as evaluated by a non-targeted gas chromatography–mass spectrometry technique [267,268,269]. Pharmacometabolomics in animal models have also been utilized to test the efficacy of psychiatric treatments, including long-term effects and adverse events [266]. In humans, the study of metabolomics has also begun. In the latest metabolomic study in patients with MDD, 23 metabolites were significantly lower in plasma when compared with non-depressed individuals, with the highest differences being reported for phosphoethanolamine, which is crucial for CNS assembly [270]. In a study of more than 5000 and 10,000 patients with depression and healthy controls, respectively, a total of 230 metabolites were analyzed in a proton nuclear magnetic resonance metabolomic platform [271]. The authors highlighted that the lipid metabolite profile, including apolipoprotein B, very low-density lipoprotein cholesterol, triglycerides, diglycerides, total and monounsaturated fatty acids, fatty acid chain length, glycoprotein acetyls, Tyr, isoleucine, high-density lipoprotein cholesterol, acetate, and apolipoprotein A1 are strongly associated with the depression phenotype, and that this lipid fingerprint is shared with common cardiovascular diseases.

**Figure 2:**
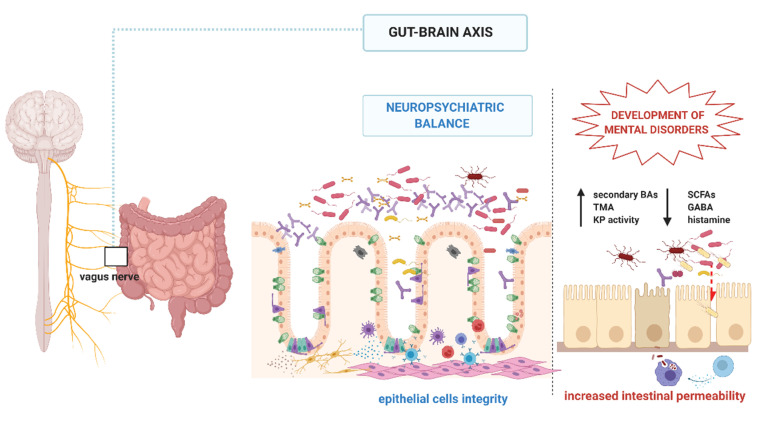
The role of the gut microbiota and the gut–brain axis in the development of mental disorders. BAs, bile acids; TMA, trimethylamine; KP, kynurenine pathway; GABA, gamma–amino butyric acid; SCFAs, short-chain fatty acids. Created with Biorender.com.

As GBA were shown to influence the CNS structure and function, targeted metabolomic analyses aimed to look for microbially-produced molecules [53,127,128,272,273]. In future, the research could be focused on newly discovered pathways involved in the CNS pathology. Consequently, we are striking more intensively to determine one’s risk towards the disease, create personalised intervention, and predict response to therapy (Figure 2).

## 12. Conclusions and Future Perspectives

In summary, it should be emphasized that GBA exists and plays a crucial role in the human body by regulating the neuropsychiatric balance. Notably, the communication is performed via the vagal nerve and is also based on molecules transmitted through the circulatory system. In this context, the most significant molecules are bacterial neurotransmitters, SCFAs, tryptophan metabolites, BAs, and *L*-carnitine/choline metabolites. The imbalance in the production of these molecules has been strongly associated with the development of multiple mental disorders that alter the gut microbiota composition. This is the up to date review that highlights the association between gut bacterial metabolites and mental illnesses. The most important limitation of this review was our non-systematic approach to the literature search, which could have resulted in a non-exhaustive analysis of the available evidence. The choice of this scoping review approach was based on limited numbers of clinical studies in humans. Therefore, future studies (especially clinical) should focus on compositional and functional analysis of microbiota in order to create potent interventional strategies, aiming to improve skewed pathways. To fully understand the role of host–microbe–metabolome interactions in mental illness, novel studies should utilize validated metagenomic and metabolomic analyses. As the microbial production of the metabolites is strain-specific, this kind of experimental evaluation could lead to the discovery of new drugs and molecules, capable of affecting certain metabolic pathways involved in the pathogenesis of common mental diseases.

## Figures and Tables

**Table 1:** Genera and species with potential for neurotransmitter synthesis [39,64,65]. GABA—gamma–amino butyric acid.

Dopamine	Noradrenaline	Serotonin	GABA	Histamine
*Bacillus cereus*	*Bacillus mycoides*	*Escherichia coli* (K-12)	*Bifidobacterium adolescentis*	*Citrobacter freuiindii*
*Bacillus mycoides*	*Bacillus subtilis*	*Hafnia alvei*	*Bifidobacterium angulatum*	*Enterobacter* spp.
*Bacillus sybtilis*	*Escherichia coli* (K-12)	*Klebsiella pneumoniae*	*Bifidobacterium dentium*	*Hafnia alvei*
*Escherichia coli*	*Proteus vulgaris*	*Lactobacillus plantarum*	*Bifiobacterium infantis*	*Klebsiella pneumoniae*
*Escherichia coli* (K-12)	*Serratia marcescens*	*Lactobacillus lactis* subsp. *Cremoris* (MG 1363)	*Lactobacillus brevis*	*Lactobacillus plantarum*
*Hafnia alvei*	*Staphylococcus* spp.	*Morganella morganii*	*Lactobacillus buchneri*	*Lactobacillus hilgardii*
*Klebsiella pneumoniae*		*Streptococcus thermophilus* (NCFB2392)	*Lactobacillus paracasei* NFRI	*Lactobacillus lactis*
*Morganella morganii*		*Candida*	*Lactobacillus plantarum*	*Morganella morganii*
*Proteus vulgaris*		*Enterococcus*	*Lactobacillus reuteri*	*Oenococcus oeni*
*Serratia marcescens*			*Lactobacillus rhamnosus*	*Pediococcus parvulus*
*Staphylococcus aureus*			*Lactobacillus delbrueskii*	*Streptococcus thermophiles*
			*Monascus purpureus*	
			*Streptococcus salivarius*	

**Table 2:** Kynurenine (KP) intermediates in psychiatric diseases [154,164].

Entity	Biological Material	KP Intermediate	Concentration Compared with Controls
Anxiety	Plasma/serum	KYN	↑
Major depression	Plasma/serum	TRP	↓
		KYN	↓
		KYNA	↓
		KYN/TRP	↑
Schizophrenia	CSF	KYN	↑
		KYNA	↑
ADHD	Serum	KYNA	↓
		TRP	↑
		KYN	↑
ASD	Serum	KYNA	↓
		KYN/KYNA	↑
		TRP	↓

ADHD, attention deficit hyperactivity disorder; ASD, autism spectrum disorder; CSF, cerebrospinal fluid; KYN, kynurenine; KYNA, kynurenic acid; TRP, tryptophan.
